# The emerging roles of long noncoding RNAs in lymphatic vascular development and disease

**DOI:** 10.1007/s00018-023-04842-4

**Published:** 2023-07-06

**Authors:** Konstantin I. Ivanov, Olga V. Samuilova, Andrey A. Zamyatnin

**Affiliations:** 1 Research Center for Translational Medicine, Sirius University of Science and Technology, Sochi, Russian Federation; 2 Department of Microbiology, University of Helsinki, Helsinki, Finland; 3 Department of Biochemistry, Sechenov First Moscow State Medical University, Moscow, Russian Federation; 4 HSE University, Moscow, Russian Federation; 5 Faculty of Bioengineering and Bioinformatics, Lomonosov Moscow State University, Moscow, Russian Federation; 6 Belozersky Institute of Physico-Chemical Biology, Lomonosov Moscow State University, Moscow, Russian Federation; 7 Faculty of Health and Medical Sciences, University of Surrey, Guildford, UK

**Keywords:** lncRNA, Lymphatics, Regulation of gene expression, Lymph node metastasis (LNM), Lymphatic vessels, Lymphatic endothelial cells (LECs), Gene regulation, Long non-coding RNA

## Abstract

Recent advances in RNA sequencing technologies helped uncover what was once uncharted territory in the human genome—the complex and versatile world of long noncoding RNAs (lncRNAs). Previously thought of as merely transcriptional “noise”, lncRNAs have now emerged as essential regulators of gene expression networks controlling development, homeostasis and disease progression. The regulatory functions of lncRNAs are broad and diverse, and the underlying molecular mechanisms are highly variable, acting at the transcriptional, post-transcriptional, translational, and post-translational levels. In recent years, evidence has accumulated to support the important role of lncRNAs in the development and functioning of the lymphatic vasculature and associated pathological processes such as tumor-induced lymphangiogenesis and cancer metastasis. In this review, we summarize the current knowledge on the role of lncRNAs in regulating the key genes and pathways involved in lymphatic vascular development and disease. Furthermore, we discuss the potential of lncRNAs as novel therapeutic targets and outline possible strategies for the development of lncRNA-based therapeutics to treat diseases of the lymphatic system.

## Introduction

About three quarters of the human genome can be transcribed [1], giving rise to millions of transcripts, most of which appear nonfunctional [2]. The remaining tens of thousands of functional transcripts correspond to either canonical protein-coding genes (PCGs) or their noncoding counterparts, which are often involved in the regulation of PCG expression. Some protein-coding RNAs are bifunctional and also possess noncoding functions and vice versa [3]. Depending on their size, noncoding functional transcripts (excluding ribosomal RNAs and transfer RNAs) can be classified into two broad and heterogeneous groups—small noncoding RNAs (sncRNAs) and long noncoding RNAs (lncRNAs). Defined as non-protein-coding transcripts longer than of 200 nucleotides (an arbitrary threshold, which was recently recommended to be raised to 500 nucleotides [4]), lncRNAs share many features with mRNAs. Like mRNAs, lncRNAs are predominantly transcribed by RNA polymerase II [5, 6], are often capped and polyadenylated, and undergo splicing [7]. Once dismissed as part of transcriptional “noise”, lncRNAs have made a remarkable comeback in recent years, providing a whole new layer of complexity to gene regulation. Despite lacking protein-coding potential, lncRNAs exert diverse gene regulatory functions through a variety of molecular mechanisms at the transcriptional, post-transcriptional, translational and post-translational levels [8–10]. Although these mechanisms typically involve lncRNA interactions with functional biomolecules like nucleic acids or proteins, the very process of lncRNA transcription may itself regulate gene expression [11]. The versatile regulatory functions of lncRNAs are being increasingly appreciated in almost every aspect of human physiology and disease [12] including developmental processes [13, 14], cell cycle [15], differentiation [16], metabolism [17], hematopoiesis [18], X-chromosome inactivation [19], stress response [20], aging [21], apoptosis [22], inflammation [23], viral infection [24] and cancer [25, 26].

The subcellular localization of an lncRNA largely determines its biological function. LncRNAs may reside in various subcellular compartments including the nucleus, cytosol, endoplasmic reticulum and mitochondria [27]. Furthermore, lncRNAs may be secreted from the cell in exosomes or other extracellular vesicles (EVs). A number of lncRNAs localize in different subcellular compartments and may have multiple functions depending on their subcellular localization. Nuclear lncRNAs typically regulate transcriptional programs through chromatin interactions and remodeling [28, 29], often serving as scaffolds for multiprotein transcriptional and epigenetic complexes. However, nuclear lncRNAs can also perform a variety of non-chromatin-related functions such as organization of nuclear paraspeckles [20], processing of preribosomal RNA in the nucleolus [30] and regulation of alternative splicing [31]. Cytoplasmic lncRNAs, on the other hand, are mainly involved in the post-transcriptional regulation of gene expression. For example, they may interact with protein-coding mRNAs to alter their translation and/or stability [32–34] or act as molecular “sponges”, also known as competing endogenous RNAs (ceRNAs), to sequester microRNAs (miRNAs) from their mRNA targets [35, 36]. MiRNAs are not the only molecules that can be “sponged” in this manner: proteins may also become sequestered through their interaction with lncRNAs [37]. In addition, protein-lncRNA interactions may participate in the scaffolding of protein–protein or protein-nucleic acid complexes [38]. Yet another function of lncRNAs in the cytoplasm is to mediate signal transduction pathways by influencing protein post-translational modifications [39] or serving as stabilizing scaffolds for signal transduction proteins [40].

In addition to subcellular localization, cell lineage specificity (or the lack of it) is another important influence on the function of lncRNAs. Certain lncRNAs are specifically expressed or enriched in particular cell types, defining their phenotypes. For example, the lineage-specific lncRNA *NeST* (nettoie Salmonella pas Theiler’s) is induced in type 1 T helper (Th1) cells, but not in type 2 T helper (Th2) cells, and regulates the expression of interferon-gamma via an epigenetic mechanism [41, 42]. Another example is spliced-transcript endothelial-enriched lncRNA (*STEEL*), which is enriched in endothelial cells and activates a pro-angiogenic transcriptional program [43]. On the other hand, there are lncRNAs that are widely expressed across almost all cell types such as the metastasis-associated lung adenocarcinoma transcript 1 (*MALAT1*) [44]. Some lncRNAs are associated with certain diseases such as coronary artery disease [45], or respond to environmental stimuli such as hypoxia [46]. Overall, lncRNAs are extremely diverse, both functionally and spatially within the cell, and there is hardly any physiological or pathophysiological process that is not affected by them to some degree. In this review, we will highlight the lncRNAs that are involved in lymphatic vascular development and disease.

The lymphatic vascular system is a unidirectional network of lymphatic capillaries and collecting lymphatic vessels lined by lymphatic endothelial cells (LECs), which is present in most of the body’s organs. The lymphatic vasculature drains interstitial fluid from tissues and returns it to blood circulation in the form of lymph. In addition to its central role in maintaining body fluid homeostasis [47], the lymphatic vasculature transports antigens and immune cells from peripheral tissues to lymph nodes (LNs), thereby contributing to immune surveillance [48–50]. Other tissue- and organ-specific functions of lymphatic vessels include dietary fat absorption in the intestine [51], antigen storage and presentation in lymphoid tissues [48], and outflow of cerebrospinal fluid in the cranial and spinal compartments of the central nervous system [52–56]. Structural and functional abnormalities of the lymphatic vasculature are associated with multiple diseases including lymphedema (accumulation of lymph in soft tissues) [57, 58], metabolic diseases such as obesity [59, 60] and diabetes [61, 62], chronic inflammation [63, 64], cardiovascular disease [65, 66], atherosclerosis [67–69], neurodegenerative diseases [70–73], glaucoma [74, 75] and Crohn’s disease [50]. Finally, the process of lymphatic vessel formation and expansion, termed lymphangiogenesis, plays an essential role in cancer progression and metastasis [49, 50, 76–79].

The present review does not attempt to cover the whole spectrum of molecular, cellular and morphological mechanisms underlying physiological and pathological lymphangiogenesis, as they have been extensively reviewed elsewhere [49, 50, 80–82]. Instead, we focus only on those molecular players and pathways for which regulatory mechanisms involving lncRNAs have been identified. Because the field is rapidly changing and new data constantly becomes available, the review does not aim at full coverage of all the existing literature. Nevertheless, we will discuss the most important findings showing how lncRNAs affect lymphatic vascular development, physiology and disease, and outline potential therapeutic approaches targeting lncRNAs for the amelioration of lymphatic vascular pathologies, including lymphedema and cancer metastasis.

## *LETR1*: lymphatic vascular lineage-specific lncRNA

Lymphatic endothelial transcriptional regulator lncRNA 1 (*LETR1*), also known as *LINC01197*, was identified by Ducoli et al. [83] and represents the first, and so far only, example of LEC-specific lncRNA. The principal difference between *LETR1* and the other lncRNAs discussed below is that *LETR1* is specifically expressed in the lymphatic but not in the blood vessel endothelium, suggesting a unique, lineage-specific role in regulating LEC differentiation. Indeed, Ducoli et al. found that *LETR1* acts as gatekeeper of the LEC transcriptome by modulating the expression of essential proliferation-related genes such as the tumor-suppressor transcription factor *KLF4* [84] and genes involved in endothelial cell migration such as the secreted semaphorin *SEMA3C* [85, 86]. They also demonstrated that, consistent with the predominantly nuclear localization of *LETR1* in LECs, *LETR1* regulates gene expression through a chromatin-based epigenetic mechanism. This mechanism involves *LETR1* being recruited to DNA regions near its target genes and interacting with the nucleosome remodeling factor RBBP7, a component of several histone deacetylase (HDAC) complexes including mSin3, NuRD and CoREST [87]. Another epigenetic complex containing RBBP7 is polycomb repressive complex 2 (PRC2), which is best known as an epigenetic “writer” of histone methylation associated with transcriptional repression [88]. Taken together, the findings of Ducoli et al. indicate that *LETR1* regulates the expression of lymphatic lineage-specific genes by acting as a scaffold for epigenetic protein complexes [83]. Furthermore, *LETR1* is the first bona fide lncRNA that could potentially serve as a lymphatic-specific biomarker.

While most of the lncRNAs discussed in this review are involved in tumor lymphangiogenesis and lymphatic metastasis, *LETR1* is the first example of an lncRNA with a role in normal lymphatic vascular development. Therefore, the discovery of *LETR1* marked a major paradigm shift away from the focus on the oncogenic roles of lymphatic-associated lncRNAs towards a broader consideration of the roles of lncRNAs in both physiological and pathological lymphangiogenesis.

## LncRNAs as regulators of the lymphangiogenic growth factor VEGF-C

Paracrine signaling by vascular endothelial growth factor C (VEGF-C) plays a central role during lymphatic vascular development [89]. VEGF-C is a secreted ligand of vascular endothelial growth factor receptor-3 (VEGFR-3), the mitogenic tyrosine kinase receptor that drives LEC proliferation and migration [80, 90, 91]. To activate VEGFR-3, VEGF-C must first undergo stepwise proteolytic processing by several proteases to generate the mature form of the protein [92, 93]. Another receptor activated by the mature form of VEGF-C is the vascular endothelial growth factor receptor-2 (VEGFR-2) [90], which is also involved in lymphangiogenesis, albeit presumably in a more limited manner [80]. The VEGF-C-mediated activation of the VEGFR-3 signaling pathway is not only essential for lymphangiogenesis [94], but also plays a role in lymphatic vessel remodeling and homeostasis [95]. In the context of cancer, overexpression of VEGF-C induces the formation and remodeling of lymphatic vessels within and around primary tumors [96]. In addition, the aberrant activation of VEGFR-3 signaling by tumor-derived VEGF-C promotes metastatic spread of tumor cells via the lymphatics [96, 97]. Moreover, VEGF-C secreted by primary tumors stimulates lymphangiogenesis in the draining LNs even before metastasis occurs [98], inducing a permissive “lymphovascular niche” to ensure successful colonization and long-term survival of metastatic cells at the secondary site [99].

A growing number of lncRNAs have been shown to function as regulators of VEGF-C expression. For example, the lncRNA *VEGFC-LNC* was found to upregulate VEGF-C expression in human umbilical vein endothelial cells (HUVECs) [100], suggesting a role for this lncRNA in the activation of VEGF-C signaling under non-diseased physiological conditions. However, most lncRNAs implicated in VEGF-C regulation have been identified in cancers, where they either promote or inhibit tumor lymphangiogenesis and lymphatic metastasis. Some of these lncRNAs and their mechanisms of action are discussed below.

### *BLACAT2*

**Fig. 1:**
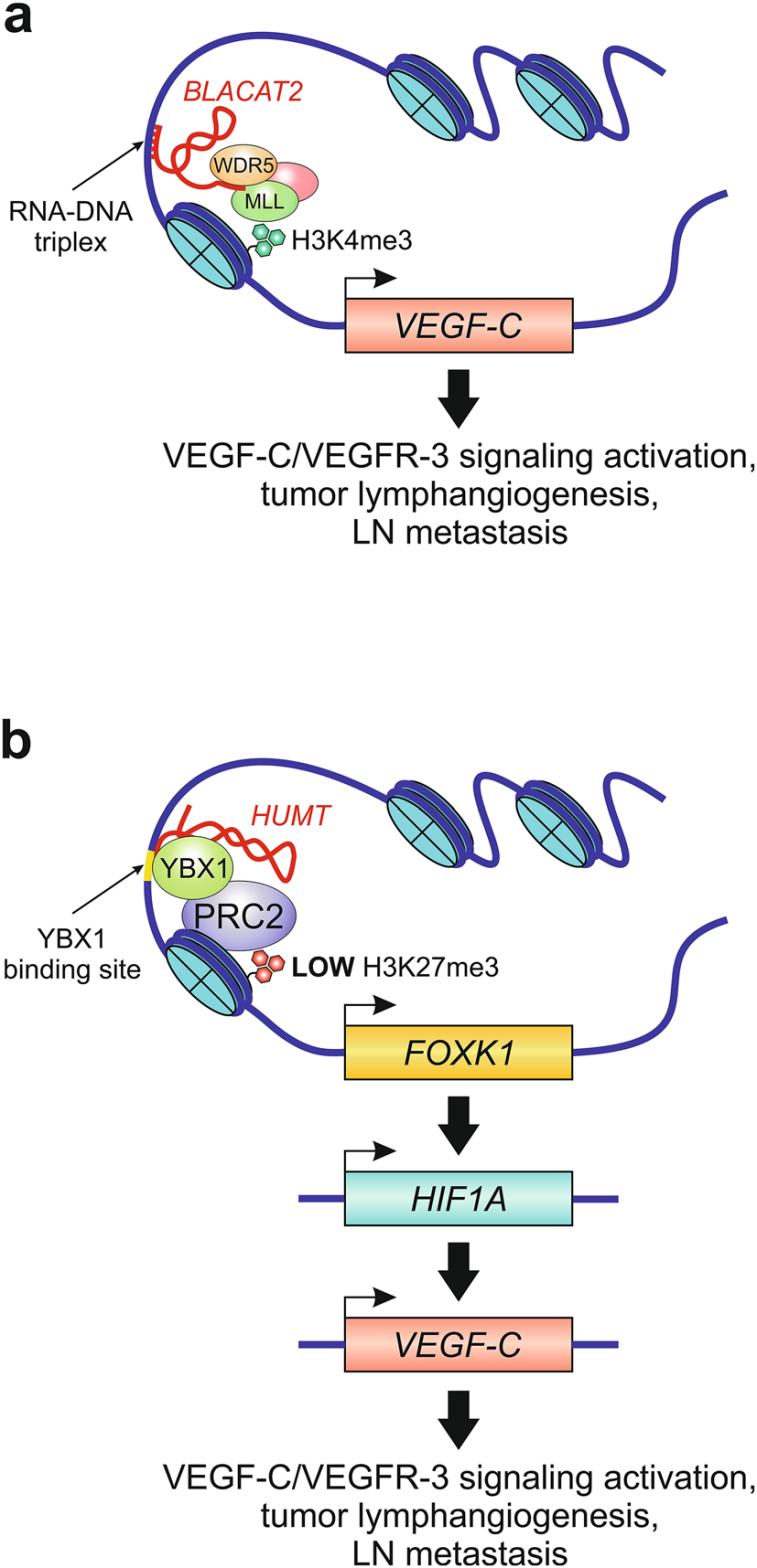
Representative lncRNA-mediated transcriptional and epigenetic mechanisms regulating the expression of the lymphangiogenic growth factor VEGF-C. **a** In the nucleus of bladder cancer cells, lncRNA *BLACAT2* interacts with the core subunit WDR5 of the histone H3 lysine 4 (H3K4) methyltransferase complex and guides it to the *VEGF-C* promoter by forming a RNA–DNA triplex with the promoter sequence. The promoter H3K4 trimethylation (H3K4me3) by the catalytic subunit MLL (mixed lineage leukemia) of the methyltransferase complex drives epigenetic activation of *VEGF-C* transcription, which leads to the activation of the VEGF-C signaling pathway, thereby inducing tumor lymphangiogenesis and lymphatic metastasis. Based on He et al. [102]. **b** In triple-negative breast cancer cells, the nuclear pool of lncRNA *HUMT* activates *FOXK1* transcription by recruiting the Y-box transcription factor YBX1 to the *FOXK1* promoter. YBX1 is a known inhibitor of the histone methyltransferase activity of the polycomb repressive complex 2 (PRC2). Therefore, the YBX1-mediated inhibition of H3K27 trimethylation (H3K27me3) at the *FOXK1* promoter may further contribute to the activation of *FOXK1* transcription. FOXK1 subsequently activates the expression of the hypoxia-inducible transcription factor HIF-1a, which in turn activates the expression of VEGF-C. Based on Zheng et al. [134]

The bladder cancer-associated transcript 2 (*BLACAT2*), also known as *LINC00958*, was initially identified by Seitz et al. [101] as a candidate oncogene in bladder cancer and is regarded as one of the first examples of an lncRNA promoting VEGF-C-induced tumor lymphangiogenesis and lymphatic metastasis. He et al. [102] found that *BLACAT2* is markedly overexpressed in metastatic bladder cancer, and its overexpression is positively correlated with LN metastasis and poor prognosis. They also showed that overexpression of *BLACAT2* promotes lymphangiogenesis and lymphatic metastasis in animal models. Furthermore, they suggested that *BLACAT2* exerts its oncogenic effect through an epigenetic mechanism that upregulates the expression of VEGF-C (Fig. 1a). In this mechanism, *BLACAT2* forms an RNA–DNA triplex with the *VEGF-C* promoter and recruits WD repeat-containing protein 5 (WDR5), a core component of the histone H3 lysine 4 (H3K4) methyltransferase complexes [103], to epigenetically promote *VEGF-C* transcription through WDR5-mediated H3K4 methylation. Thus *BLACAT2* promotes tumor lymphangiogenesis and lymphatic metastasis by epigenetically upregulating VEGF-C expression and pathologically activating the VEGFR-3 signaling pathway [102].

### *LNMAT1*

Lymph node metastasis-associated transcript 1 (*LNMAT1*), alternatively named *DUXAP9* or *LINC01296*, was identified as a candidate oncogene in bladder cancer in the same study by Seitz et al. [101] that discovered the oncogenic properties of *BLACAT2*. Chen et al. [104] showed that *LNMAT1* is a nuclear-enriched lncRNA that is overexpressed in LN-positive bladder cancer and is associated with lymphatic metastasis and poor prognosis. Furthermore they demonstrated that, consistent with its oncogenic role in human bladder cancer, *LNMAT1* overexpression induces lymphangiogenesis and lymphatic metastasis in animal models. According to Chen et al., the function of *LNMAT1* in metastatic bladder cancer revolves around epigenetic activation of the small chemokine CCL2, which manifests itself in the deposition of H3K4 trimethyl activating marks at the *CCL2* promoter region. Mechanistically, *LNMAT1* forms a DNA-RNA triplex with the *CCL2* promoter and interacts with the heterogeneous nuclear ribonucleoprotein L (HNRNPL) known for its role in transcriptional regulation [23]. By this means, *LNMAT1* recruits HNRNPL to the *CCL2* promoter and activates its transcription in the nucleus of bladder cancer cells. CCL2 activation and subsequent secretion by bladder cancer cells in turn upregulates VEGF-C expression in tumor-associated macrophages, promoting lymphangiogenesis and lymphatic metastasis [104]. It should be noted, however, that despite the established role of CCL2 in developmental and tumor-associated lymphangiogenesis [105], the exact molecular mechanism linking CCL2 activation with the enhanced VEGF-C expression remains to be elucidated.

### *BCYRN1*

Brain cytoplasmic RNA 1 (*BCYRN1*), also known as *LINC00004*, is another example of an oncogenic lncRNA that promotes lymphatic metastasis through the activation of VEGF-C expression. Zheng et al. [106] found that *BCYRN1* was significantly enriched in urinary exosomes from patients with bladder cancer compared to healthy controls. Furthermore, elevated levels of exosomal *BCYRN1* were associated with increased lymphatic metastasis, decreased survival and poor prognosis in these patients. The authors proposed a dual mechanism of action for *BCYRN1* in bladder cancer. On one hand, *BCYRN1* activates the Wnt/β‐catenin signaling pathway to promote the secretion of VEGF‐C. To achieve this, *BCYRN1* forms a DNA‐RNA triplex with the promoter of the *WNT5A* gene and, in association with the RNA‐binding protein HNRNPA1, induces its expression via H3K4 trimethylation [106]. Since WNT5A is a Wnt ligand [107], its overexpression activates the Wnt/β‐catenin signaling pathway, increasing the expression and secretion of VEGF‐C, which is a known Wnt target gene [108, 109]. On the other hand, Zheng et al. showed that *BCYRN1* plays an important role in exosome‐mediated communication between bladder cancer cells and LECs. They found that *BCYRN1* is transported via exosomes from bladder cancer cells to LECs, where it stabilizes *VEGFR-3* mRNA by interacting with its 3′‐UTR. This results in the increased expression of VEGFR-3 on the surface of LECs, making the cells more sensitive to VEGF-C. Thus, *BCYRN1* simultaneously increases both VEGFR-3 expression in LECs and VEGF-C secretion from bladder cancer cells, creating a feedforward loop that promotes tumor lymphangiogenesis and LN metastasis in bladder cancer through amplified VEGF‐C/VEGFR-3 signaling [106].

### *VESTAR*

*VEGF-C* mRNA stability-associated lncRNA (*VESTAR*), otherwise known as *LINC00638*, was identified by Wang et al. [110] in a chromosomal region frequently amplified in esophageal squamous cell carcinoma. They found that *VESTAR* is overexpressed in esophageal and several other digestive system cancers, including cancers of the liver, stomach, and colon. Furthermore, they showed that *VESTAR* overexpression in esophageal squamous cell carcinoma tissues is accompanied by a change in its subcellular localization from the nucleus to the cytoplasm, suggesting a nuclear export mechanism. Indeed, *VESTAR* interacts with nuclear RNA export factor 1 (NXF1) and its most potent export adaptor SRSF3 [111] to facilitate its export from the nucleus. Notably, nuclear export of *VESTAR* correlates with regional LN metastasis, and this correlation was attributed to the *VESTAR*-mediated pathological activation of VEGF-C signaling [110]. The activation is due to an increase in *VEGF-C* mRNA stability caused by its direct interaction with *VESTAR* in the cytoplasm of esophageal cancer cells. In addition, *VESTAR* interacts with the RNA-binding protein HuR, enhancing its previously reported stabilizing effect on *VEGF-C* mRNA [112]. Consequently, overexpressed cytoplasmic *VESTAR* functions in association with HuR as a *VEGF-C* mRNA stabilizing factor, thereby promoting tumor-associated lymphangiogenesis and LN metastasis via aberrant activation of the VEGF-C signaling pathway [110].

### *ASLNC07322*

SMAD family member 4 (SMAD4) is a critical component of the TGF-β pathway that acts as a tumor suppressor in several cancers, including pancreatic, bile duct and colon cancer [113]. Li et al. [114] demonstrated that SMAD4 represses VEGF-C expression in colon cancer by activating the transcription of the gene encoding miR-128-3p, a microRNA targeting the 3′ UTR region of *VEGF-C* mRNA. Furthermore, they found that the repression of VEGF-C by miR-128-3p could be relieved by the oncogenic lncRNA *ASLNC07322*, which acts as the miR-128-3p sponge. Thus, *ASLNC07322* overexpression abrogates the tumor-suppressive effect of SMAD4, leading to the uncontrolled expression of VEGF-C, which in turn promotes tumor lymphangiogenesis and lymphatic metastasis [114].

### *AFAP1-AS1*

A similar mechanism involving the activation of VEGF-C expression via microRNA sponging is employed by the lncRNA *AFAP1-AS1,* which stands for actin filament-associated protein 1 antisense RNA 1. *AFAP1-AS1* is an oncogenic lncRNA [115] that, according to a recent study by Xia et al. [116], acts as a sponge for miR-27b-3p in cervical cancer cells, sequestering it away from its target *VEGF-C*. The study suggested that the derepression of VEGF-C by *AFAP1-AS1* could be responsible for promoting lymphatic metastasis and enhancing cervical cancer stemness.

### *DANCR*

Differentiation antagonistic non-protein coding RNA (*DANCR*), also called *ANCR* or *AGU2*, plays an important role in the progression, invasion and metastasis of several cancers, including cervical, pancreatic and bladder cancers [117–119]. In the case of bladder cancer, this lncRNA has been shown to promote lymphatic metastasis through sponging of miR-335 and derepression of its target gene, *VEGF-C* [120]. In this way, *DANCR* overexpression in bladder cancer leads to a pathological activation of pro-lymphangiogenic VEGF-C signaling, thereby promoting tumor lymphangiogenesis and LN metastasis.

### *MFSD4A-AS1*

A recent study by Liu et al. [121] provides yet another example of how lncRNAs can regulate VEGF-C expression through miRNA sponging. The study found that the lymphatic node metastatsis-related lncRNA *MFSD4A-AS1* is upregulated in papillary thyroid cancer (PTC) tissues with LN metastasis. Furthermore, the study showed that *MFSD4A-AS1* promotes lymphangiogenesis and lymphatic metastasis in PTC by acting as a sponge for miR-30c-2-3p and miR-145-3p to induce VEGF-C expression via the ceRNA mechanism [121].

### *HNF1A-AS1*

Hepatocyte nuclear factor 1 homeobox A antisense RNA 1 (*HNF1A-AS1*), also known as *HAS1*, is a tumor-associated lncRNA with an established role in the development and progression of many cancers, including those of the head and neck, breast, lung, bone, liver, colon, esophagus, bladder, and cervix [122]. Liu et al. [123] reported that *HNF1A-AS1* overexpression correlates with LN metastasis in gastric cancer patients and promotes metastasis of gastric cancer in a xenograft mouse model. The authors attributed this effect to the ability of *HNF1A-AS1* to induce lymphangiogenesis through the activation of the PI3K/AKT signaling pathway. PI3K/AKT signaling is one of the most frequently dysregulated pathways in cancer [124], and its aberrant activation promotes tumor lymphagiogenesis and lymphatic metastasis via increased expression and secretion of VEGF-C [125]. According to Liu et al., *HNF1A-AS1* acts as a ceRNA for miR-30b-3p to upregulate the expression of the *PIK3CD* gene, which encodes the delta isoform of the catalytic subunit of phosphoinositide 3-kinase (PI3K-delta), a key component of the PI3K/AKT signaling pathway and a known oncogene [126]. Thus, the *HNF1A-AS1*-mediated upregulation of PI3K-delta activates PI3K/AKT signaling in gastric cancer cells, inducing VEGF-C secretion and ultimately promoting tumor lymphangiogenesis and lymphatic metastasis [123].

### *circNFIB1*

The circular lncRNA *NFIB1* (*hsa_circ_0086375*) differs from the other lncRNAs discussed here in that it has anti-lymphangiogenic properties and is downregulated in pancreatic ductal adenocarcinoma (PDAC) patients with LN metastasis [127]. Circular RNAs (circRNAs) are characterized by a covalently closed circular structure produced through a non-canonical form of splicing called “back-splicing” [128]. Unlike linear lncRNAs, circular RNAs lack a 5′ cap and a 3′ polyadenylated tail, making them more resistant to RNase-mediated degradation [129]. Although the majority of circRNAs are thought to be the products of splicing errors [130], some do have functional roles in development and disease. One such is *circNFIB1*, which was shown by Kong et al. [127] to inhibit lymphangiogenesis in vitro and suppress LN metastasis of PDAC in a mouse model. Circular lncRNAs typically act as post-transcriptional regulators through miRNA sponging [131–133], and *circNFIB1* is no exception. According to Kong et al., *circNFIB1* functions as a sponge for the oncogenic miR-486-5p in the cytoplasm of PDAC cells to derepress its target, the regulatory subunit of phosphoinositide 3-kinase (PIK3R1). The derepression of PIK3R1, in turn, leads to the downregulation of VEGF-C expression via inhibition of the PI3K/Akt pathway. Overall, the findings of Kong et al. indicate that *circNFIB1* is an anti-lymphangiogenic lncRNA that suppresses lymphangiogenesis and LN metastasis in PDAC via the miR-486-5p/PI3KR1/VEGF-C axis [127].

### *HUMT*

*LINC00857*, better known as *HUMT*, which stands for lncRNA highly upregulated in metastatic triple-negative breast cancer (TNBC), is yet another lncRNA that regulates, albeit indirectly, the expression of VEGF-C in cancer. As its name implies, *HUMT* is highly expressed in TNBC [134], which is the most malignant subtype of breast cancer with the highest lymphatic metastatic potential [135]. Using a combination of bioinformatic and biochemical approaches, Zheng et al. [134] demonstrated that in the nucleus of TNBC cells, *HUMT* recruits the Y-box transcription factor YBX1, a PRC2 interactor and inhibitor of H3K27me3 [136], to the promoter region of the forkhead box K1 transcription factor (FOXK1) and activates its transcription [134] (Fig. 1b). FOXK1 is a known inducer of the hypoxia-inducible transcription factor 1 alpha (HIF-1α) [137], which in turn activates the expression of VEGF-C [138–140]. Thus *HUMT* promotes tumor-induced lymphangiogenesis and lymphatic metastasis in TNBC by pathologically activating the VEGF-C signaling pathway via the FOXK1/HIF-1α axis [134].

## LncRNA-mediated regulation of pro-lymphangiogenic VEGF-A signaling

VEGF-A is a hypoxia-driven secreted growth factor that signals through vascular endothelial growth factor receptors 1 and 2 (VEGFR-1 and VEGFR-2) to induce proliferation, migration, sprouting, permeability and survival of endothelial cells [141]. VEGF-A belongs to the same family of proteins as VEGF-C and plays a critical role in developmental and pathological angiogenesis. In addition to its role in angiogenesis, VEGF-A signaling through VEGFR-2 is also implicated in lymphangiogenesis [80], along with VEGF-C signaling through VEGFR-3/VEGFR-2. Therefore, dysregulation of VEGF-A expression by lncRNAs may contribute to the pathogenesis of lymphatic-associated diseases including metastatic cancer. Indeed, Shi et al. [142] showed that the aberrant induction of VEGF-A by the lncRNA *HANR* (also known as *RPL13AP20*) promotes tumor lymphangiogenesis in hepatocellular carcinoma. They also found that the underlying molecular mechanism involves the *HANR*-mediated sponging of miR‐296 in hepatocellular carcinoma cells, reducing the release of miR‐296 from the cells in the form of exosomes. Consequently, the LECs are able to internalize less exosomal miR‐296, causing derepression of the miR‐296 target EAG1, a potassium channel protein known to induce HIF-1α and promote the expression of VEGF-A [143]. Thus the overexpression of *HANR* in hepatic cancer cells leads to the activation of the VEGF-A/VEGFR-2 signaling pathway in LECs, thereby promoting tumor-associated lymphangiogenesis [142]. Another example of how an lncRNA can induce tumor lymphangiogenesis by upregulating VEGF-A expression comes from the above-mentioned study on the role of *MFSD4A-AS1* in papillary thyroid cancer. In addition to demonstrating that *MFSD4A-AS1* controls VEGF-C expression through miRNA sponging, the study identified a similar mechanism regulating the expression of VEGF-A. According to this mechanism, *MFSD4A-AS1* acts as a sponge for miR-139-5p to upregulate the expression of its target gene, *VEGF-A*. This leads to pathological activation of the VEGF-A/VEGFR-2 signaling pathway, which in turn stimulates lymphangiogenesis and lymphatic metastasis in papillary thyroid cancer [121].

## *NEAT1*: an IRES-dependent translational regulator of mRNAs encoding lymphangiogenic growth factors

As one of the best-studied oncogenic lncRNAs, nuclear paraspeckle assembly transcript 1 (*NEAT1*) is known to promote metastasis of various cancers, including those of the breast, lung, thyroid gland, colon, ovary, prostate, and liver [144]. Despite the rapidly accumulating knowledge about the diverse mechanisms through which *NEAT1* exerts its oncogenic activity [144, 145], our understanding of how *NEAT1* dysregulates the key lymphangiogenic pathways to promote lymphatic metastasis is still limited. In a recent study, Godet et al. [146] identified the essential nuclear paraspeckle component *NEAT1* as a novel translational regulator which enhances the translation of fibroblast growth factor 1 (FGF-1) as well as VEGF-C and VEGF-A via internal ribosome entry sites (IRESs) in the corresponding mRNAs. Since FGF-1, VEGF-C and VEGF-A are all known inducers of lymphangiogenesis [89, 141, 147], the results of Godet et al. shed light on a possible mechanism by which *NEAT1* may activate FGF, VEGF-C or VEGF-A signaling in cancer, thereby promoting tumor lymphangiogenesis and lymphatic metastasis. Furthermore, the notion that *NEAT1* is a pro-lymphangiogenic lncRNA is also supported by an independent study demonstrating that *NEAT1* upregulates VEGF-C expression in bladder cancer by sponging its negative regulator miR-101 [148].

## Regulation of lymphangiogenic growth factor IGF-1 by *LncCCLM*

Cancer lymphatic metastasis-associated lncRNA (*LncCCLM*), also known as *RP11-7K24.3*, is an example of an lncRNA that acts as a suppressor of lymphatic metastasis. Chen et al. [149] found that *LncCCLM* is downregulated in cervical cancer tissues, and its low expression is associated with an increased risk of distant lymphatic metastasis. They also showed that *LncCCLM* decreases cervical cancer cell migration and invasion in vitro and inhibits lymphatic metastasis of cervical cancer in a mouse model. The authors proposed a mechanism for the action of *LncCCLM* in cervical cancer, according to which cytoplasmic *LncCCLM* interacts with Staufen double-stranded RNA binding protein 1 (STAU1) to promote the decay of the insulin-like growth factor 1 (*IGF-1*) mRNA. This leads to a decrease in the amount of IGF-1 protein, which is a known inducer of tumor lymphangiogenesis and lymphatic metastasis [150]. Based on these findings, Chen et al. concluded that *LncCCLM* functions as a suppressor of lymphatic metastasis in cervical cancer by inhibiting the pro-lymphangiogenic IGF-1/IGF-1R signaling pathway via the STAU1-mediated degradation of *IGF-1* mRNA [149].

## LncRNA-mediated control of PROX1, the master regulator of lymphatic differentiation and development

The prospero homeodomain transcription factor (PROX1) is the master regulator of LEC identity, initiating and maintaining the specific transcriptional program that governs LEC differentiation from a subpopulation of venous endothelial cells (VECs) [49, 82]. PROX1 serves as a specific marker of developing and adult lymphatic vasculature [151] and deregulation of its expression is linked to several lymphatic-associated diseases comprising the metabolic syndrome [62] such as hyperlipidemia, obesity and diabetes [152, 153]. In addition, PROX1 plays an important but ambivalent role in cancer [154] as either oncogene [155–158] or tumor suppressor [159–162], depending on the cancer type and context. Therefore, a better understanding of the molecular mechanisms regulating PROX1 expression not only expands our knowledge of normal lymphatic development and function, but also provides novel insights into the pathophysiology of lymphatic-associated diseases and cancer.

## *ANRIL* and *GAS5*

**Fig. 2:**
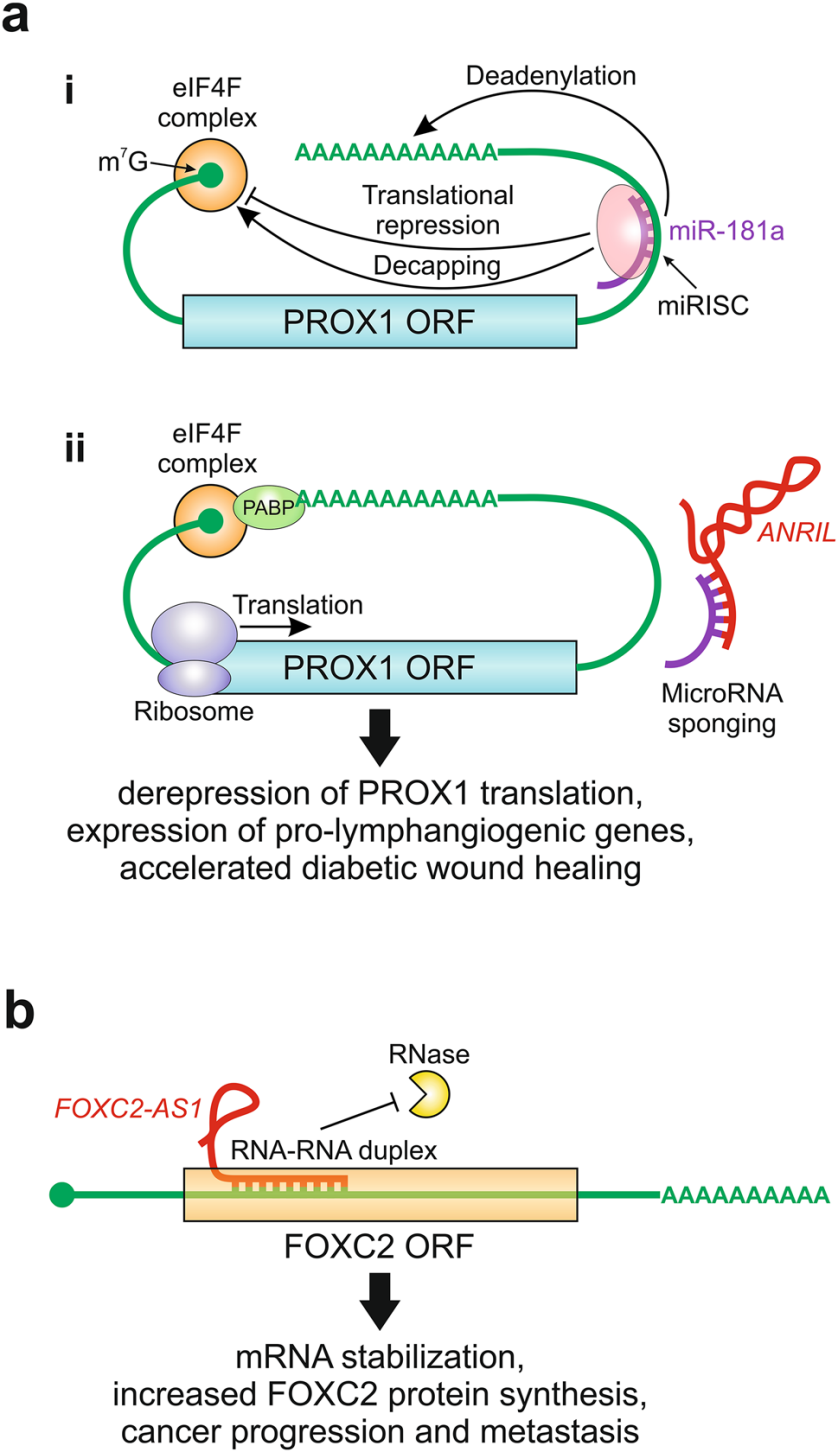
Examples of cytoplasmic mechanisms by which lncRNAs regulate key molecular players involved in lymphangiogenesis. **a** MicroRNA sponging. (i) miR-181a binds to the 3′-UTR of the *PROX1* mRNA inducing translational repression and mRNA decay. (ii) The lncRNA *ANRIL* functions as a decoy to sponge miR-181a away from *PROX1* mRNA, derepressing its translation. The increased translation of *PROX1* leads to the activation of genes that promote lymphangiogenesis, thereby accelerating diabetic wound healing. Based on He et al. [167]. **b** Regulation of mRNA stability. The natural antisense lncRNA *FOXC2-AS1* forms an RNA-RNA duplex with the *FOXC2* mRNA, stabilizing it and protecting it from RNase-mediated cleavage. The resulting aberrant increase in *FOXC2* translation promotes EMT and tumor metastasis. In a non-cancer context, the same mechanism might regulate lymphatic valve formation and collecting lymphatic vessel specialization. Based on Zhang et al. [208] and Missaglia et al. [216]. *eIF4F* eukaryotic initiation factor 4F, *m7G* methyl-7-guanosine (cap), *miRISC* microRNA-induced silencing complex, *ORF* open reading frame, *PABP* poly(A)-binding protein

The antisense noncoding RNA in the INK4 locus (*ANRIL*), also called *CDKN2B-AS1*, was first identified in patients with familial melanoma [163]. Cunnington et al. [164] subsequently showed that *ANRIL* is associated with coronary artery disease and diabetes, where its expression is downregulated. On the other hand, Sun et al. [165] found that *ANRIL* promotes lymphangiogenesis and lymphatic metastasis in colorectal cancer by upregulating the expression of VEGF-C and VEGFR-3. These findings prompted He et al. [167] to investigate whether *ANRIL* might promote lymphangiogenesis to accelerate the process of wound healing, which is impaired in diabetes [166]. Indeed, the authors showed that *ANRIL* upregulates the expression of PROX1 in LECs on a post-transcriptional level, thereby promoting lymphangiogenesis and accelerating wound healing [167]. Mechanistically, this is achieved by *ANRIL*-mediated sponging of miR‐181a (Fig. 2a), which was characterized previously as a negative regulator of PROX1 expression [168].

The lncRNA growth arrest-specific transcript 5 (*GAS5*), also known as *SNHG2*, was originally identified as a non-protein-coding tumor suppressor gene that is highly expressed during growth arrest [169]. *GAS5* is downregulated in many cancers including breast, prostate, lung and colorectal cancer, and its reduced expression correlates with poor prognosis [170]. In a non-cancer setting, *GAS5* participates in diabetic wound healing promoted by topical mevastatin [171]. As just described in the case of *ANRIL*, *GAS5* promotes lymphangiogenesis during wound healing via a PROX1-dependent mechanism. To establish this mechanism, He et al. [172] first carried out bioinformatics analysis and found that both *GAS5* and *PROX1* contain binding sites for the same microRNA, miR-217. Consequently, they showed that *GAS5* acts in a similar fashion to *ANRIL*, sponging miR-217 and thereby derepressing the expression of PROX1.

Given the similarities in the pro-lymphangiogenic mechanisms of *ANRIL* and *GAS*5, it is not surprising that both lncRNAs behave quite similarly [167, 172]. Both *ANRIL* and *GAS5* are downregulated in the skin of diabetic mice or human patients. Furthermore, treating LECs with high glucose downregulates the expression of both lncRNAs, leading to the inhibition of lymphangiogenesis. On the other hand, overexpression of *ANRIL* or *GAS5* accelerates wound healing, underscoring the prominent role of lymphangiogenesis in this process. Interestingly, the *ANRIL*- or *GAS5*-mediated sponging of their respective miRNAs upregulates the expression of not only PROX1, but also its direct target VEGFR-3 [173]. Taken together, the above findings indicate that *ANRIL* and *GAS5* effectively improve wound healing by promoting lymphangiogenesis via the PROX1/miR‐181a and PROX1/miR-217 axes, thus highlighting the importance of these two lncRNAs in the regulation of the PROX1-mediated LEC-specific transcriptional program. Therefore, both *ANRIL* and *GAS5* deserve further evaluation as potential therapeutic targets for the treatment of delayed wound healing in diabetic patients [167, 172]. Finally, it should be noted that the opposing roles of *ANRIL* and *GAS5* as tumor promoter versus tumor suppressor likely reflect the ambivalent role of PROX1 in cancer.

### *MIAT*

Another example of PROX1 regulation by miRNA sponging is provided by the lncRNA myocardial infarction‑associated transcript (*MIAT*), also known as *RNCR2* or Gomafu. *MIAT* plays an important role in development and various diseases [174] and is involved in the differentiation of mesenchymal stem cells (MSCs) into endothelial cells (ECs) [175]. In a recent study, *MIAT* was shown to promote the differentiation of adipose‑derived mesenchymal stem cells (ADMSCs) into LECs by regulating the expression of PROX1 [176]. To achieve this, *MIAT* acts as a molecular sponge of miR-495, for which a binding site has been identified in the 3′‑untranslated region of the *PROX1* mRNA. Thus, *MIAT* upregulates PROX1 expression through competitive binding to miR-495, thereby promoting the transcriptional reprogramming of ADMSCs into LECs. Since the induced differentiation of ADMSCs into LECs is emerging as a novel avenue for the treatment of lymphedema [177–179], *MIAT* has been proposed as a potential therapeutic target in this disease [176].

### *LNMAT2*

**Fig. 3:**
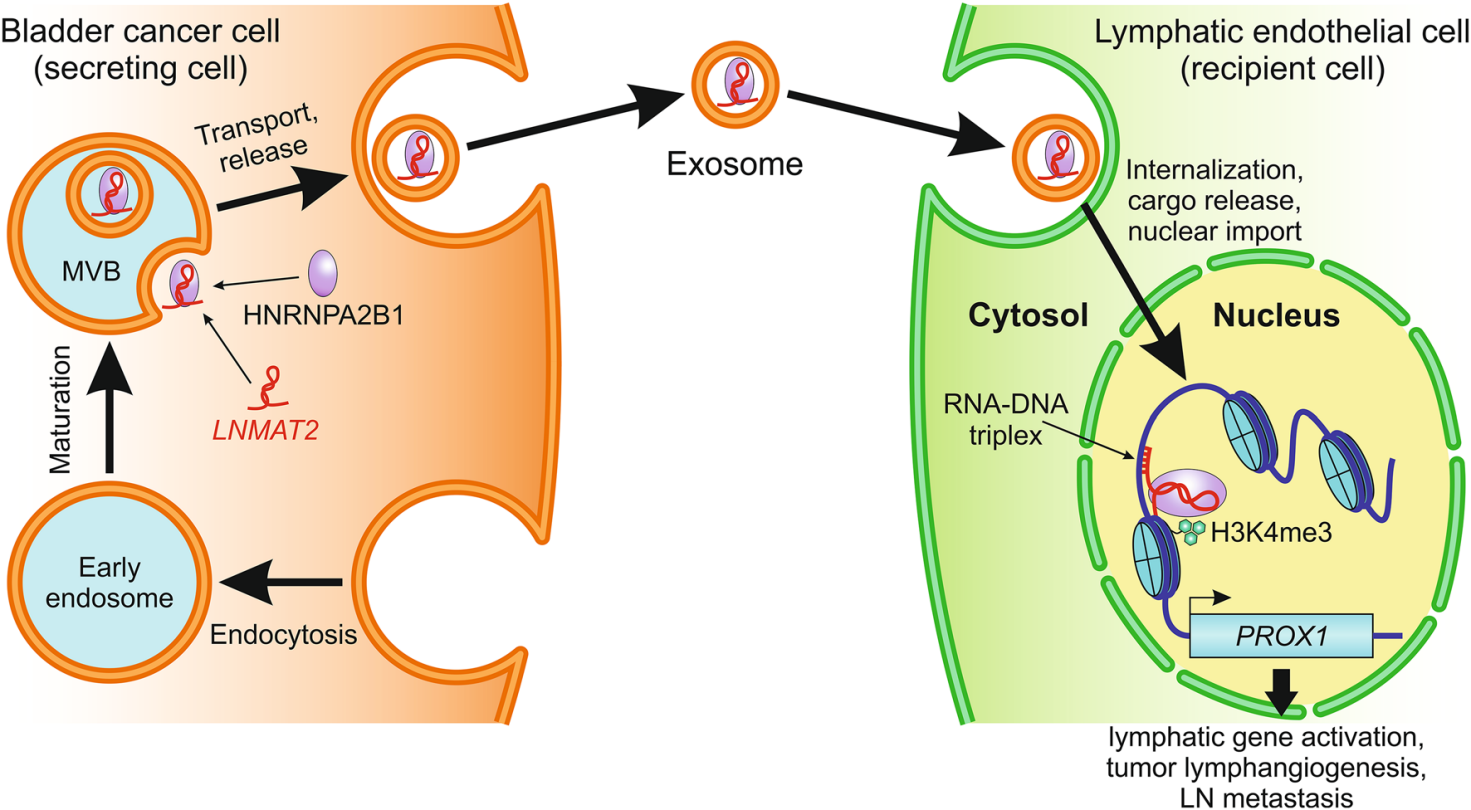
Representative mechanism of intercellular communication between cancer cells and lymphatic endothelial cells (LECs) mediated by exosomal lncRNA. The lncRNA *LNMAT2*, which is overexpressed in bladder cancer cells, contains the exo-motif GGAG recognized by the RNA binding protein HNRNPA2B1. The interaction with HNRNPA2B1 facilitates *LNMAT2* sorting into exosomes, which are formed during endosome maturation through inward membrane budding of multivesicular bodies (MVBs). The exosomes are secreted out of cancer cells and subsequently internalized by LECs. Upon entering LECs, the exosomes dissociate and their cargo translocates into the nucleus, where *LNMAT2* forms a DNA-RNA triplex with the *PROX1* promoter. The *LNMAT2*-tethered HNRNPA2B1 activates *PROX1* transcription by increasing the levels of H3K4 trimethylation (H3K4me3) in the promoter region. The epigenetically induced overexpression of PROX1 results in aberrant transcriptional reprogramming, which promotes tumor lymphangiogenesis and LN metastasis. Based on Chen et al. [180]

Yet another mechanism by which an lncRNA is able to regulate PROX1 expression has been identified in the context of bladder cancer. The lncRNA lymph node metastasis-associated transcript 2 (*LNMAT2*), otherwise known as *LINC00858*, was found by Chen et al. [180] to be overexpressed in bladder cancer cells, and its overexpression positively correlated with LN metastasis. They also showed that, in order to fulfill its lymphatic metastatic potential, the overexpressed *LNMAT2* interacts with the RNA‐binding protein HNRNPA2B1 (heterogeneous nuclear ribonucleoprotein A2/B1) and the resulting complex is released by cancer cells via exosomes (Fig. 3). The exosomes are subsequently internalized by LECs, promoting tumor-associated lymphangiogenesis and lymphatic metastasis through an epigenetic mechanism upregulating the transcription of *PROX1*. The mechanism involves the formation of a DNA-RNA triplex between *LNMAT2* and the *PROX1* promoter, thereby recruiting the *LNMAT2*-tethered HNRNPA2B1 to the promoter region and increasing its H3K4 trimethylation (H3K4me3) [180]. The resulting aberrant epigenetic activation of PROX1 induces transcriptional reprogramming, which leads to the uncontrolled expression of lymphatic genes. Interestingly, the *LNMAT2*-mediated mechanism of PROX1 activation is VEGF-C-independent, explaining why approximately 20% of bladder cancers with LN metastasis have low VEGF-C expression [181, 182].

### *PROX1-AS1*

*PROX1-AS1*, a natural antisense transcript overlapping the *PROX1* gene, is an oncogenic lncRNA that is upregulated in renal and lung cancer and positively correlates with tumor metastasis [183, 184]. However, despite these findings, the role of *PROX1-AS1* in the regulation of PROX1 during lymphatic vascular development and disease is currently unknown and requires further investigation.

## Regulation of lymphangiogenic transcription factor SOX18 by *ELNAT1*

In addition to PROX1, several other transcription factors play a critical role in the specification of endothelial cells into lymphatic lineage. One of them is SRY-Box transcription factor 18 (SOX18), which activates the expression of PROX1 [185] in cooperation with chick ovalbumin upstream promoter transcription factor 2 (COUP-TFII) [186]. Recently, an lncRNA named extracellular lymph node-associated transcript 1 (*ELNAT1*), also known as *SNHG16*, has been shown by Chen et al. [187] to epigenetically upregulate the expression of SOX18, thereby inducing tumor lymphangiogenesis and lymphatic metastasis in bladder cancer. The molecular mechanism underlying this pathological process resembles that of *LNMAT2*. In the nucleus of bladder cancer cells, *ELNAT1* forms a DNA-RNA triplex with the promoter of small ubiquitin-like modifier (SUMO)-conjugating enzyme 9 (UBC9) and, in association with the RNA‐binding protein HNRNPA1, activates the transcription of the *UBC9* gene. The overexpressed UBC9 promotes the SUMOylation of HNRNPA1 to facilitate sorting and packaging of *ELNAT1* into extracellular vesicles. The packaged vesicles are secreted by cancer cells and then internalized by LECs, where *ELNAT1* is translocated into the nucleus, once again forming a DNA-RNA triplex in association with HNRNPA1, but this time with the *SOX18* promoter. The epigenetic activation of the *SOX18* gene in LECs is evident from the increased levels of HNRNPA1-induced H3K4me3 at the promoter region. Based on these findings, Chen et al. concluded that the overexpression and subsequent secretion of *ELNAT1* from bladder cancer cells promotes tumor lymphangiogenesis and lymphatic metastasis through the upregulation of SOX18, which in turn leads to the aberrant activation of the PROX1-driven lymphatic transcriptional program [187].

## *MAPK8IP1P2* as a regulator of the Hippo pathway

The Hippo-YAP/TAZ signaling pathway plays an essential role in organ growth and tissue homeostasis [188, 189] and its dysregulation is commonly associated with cancer development and progression [190]. Increasing evidence suggests that the Hippo pathway is also critical during lymphatic vascular development. For instance, Hippo signaling negatively regulates PROX1 expression during LEC specification and sprouting [191]. In line with this, it has been proposed that the Hippo signaling effectors YAP/TAZ play a role in the PROX1/VEGFR-3 feedback loop during LEC specification and migration and lymphatic valve maturation [192].

Liu et al. [193] reported that the lncRNA *MAPK8IP1P2* was downregulated in thyroid carcinoma with lymphatic metastasis. They also found that *MAPK8IP1P2* acts as a sponge for miR-146b-3p to relieve the repression of three tumor suppressor genes involved in Hippo signaling. The first gene, *NF2*, is often mutated or inactivated in cancer [194, 195] and serves as an activator of the Hippo pathway [196, 197]. The two other genes, *RASSF1* and *RASSF5*, belong to the C-terminal Ras-association domain family (RASSF), whose members suppress tumorigenesis by binding to and regulating the upstream Hippo kinase MST1/2 [198–200]. Liu et al. showed that the *MAPK8IP1P2*-mediated upregulation of NF2, RASSF1 and RASSF5 leads to the activation of the antitumorigenic Hippo signaling pathway in thyroid cancer cells. Thus *MAPK8IP1P2* acts as an oncosuppressor lncRNA with anti-lymphangiogenic properties, inhibiting lymphatic metastasis in thyroid cancer via the activation of Hippo signaling [193].

## LncRNA-mediated control of the master regulator of lymphatic valve morphogenesis FOXC2

FOXC2 is a lymph flow-induced forkhead transcription factor which acts as a master regulator of lymphatic valve morphogenesis [201, 202]. In addition to its central role in the formation of lymphatic valves, FOXC2 also controls specialization of collecting lymphatic vessels during embryonic and postnatal development [203]. Inactivating mutations in *FOXC2* are the underlying cause of lymphedema-distichiasis syndrome (LD; OMIM 153400) characterized by late-onset hereditary lymphedema and the presence of a double row of eyelashes (distichiasis) [204, 205]. Furthermore, FOXC2 acts as a potent oncogene, promoting cancer cell proliferation and epithelial–mesenchymal transition (EMT), which is considered a key step in tumor metastasis [206].

A natural antisense transcript (NAT) overlapping the *FOXC2* gene, designated *FOXC2-AS1*, was first identified by microarray analysis in doxorubicin-resistant osteosarcoma cell lines [207]. Zhang et al. [208] found that in these cells *FOXC2-AS1* was predominantly localized in the cytoplasm in close proximity to *FOXC2* mRNA. This finding, together with the fact that *FOXC2-AS1* is fully complementary to 145 nucleotides in the first exon of *FOXC2*, prompted the authors to suggest that the two RNA molecules could interact, forming an RNA-RNA duplex. Indeed, Zhang et al. observed the formation of a double-stranded complex of *FOXC2-AS1* and *FOXC2* mRNA in the overlapping complementary sequence region. The complex formation has a stabilizing effect on *FOXC2* mRNA, protecting it from RNase cleavage, and thus leading to an increase in the FOXC2 protein synthesis (Fig. 2b). Given the well-established role of FOXC2 dysregulation in cancer, the results of Zhang et al. implicated the FOXC2 inducer *FOXC2-AS1* as a novel oncogene, whose aberrant overexpression contributes to cancer development, progression and metastasis, and correlates positively with poor prognosis [208]. The oncogenic potential of *FOXC2-AS1* has been since confirmed in several other cancers including breast, lung, skin, colorectal, gastric and prostate tumors [209–214]. In colorectal and gastric cancers, *FOXC2-AS1* utilizes a similar mechanism involving *FOXC2* mRNA stabilization [212, 213]. However, in prostate cancer cells, cytoplasmic *FOXC2-AS1* acts as a molecular sponge to sequester miR-1253 from its target *EZH2* [214], which encodes an oncogenic histone methyltransferase and catalytic subunit of PRC2 [215]. In addition to the above cytoplasmic mechanisms involving mRNA stabilization and miRNA sponging, another oncogenic mechanism of *FOXC2-AS1* action has been identified in the nucleus of non-small cell lung cancer (NSCLC) and melanoma cells. In these cells, the nuclear pool of *FOXC2-AS1* mediates epigenetic repression of the tumor suppressor gene *p15* via recruitment of PRC2 to its promoter [210, 211].

Recently experimental evidence has emerged suggesting that at least some of the above *FOXC2-AS1*-mediated mechanisms are operational in non-cancer cells. Missaglia et al. [216] detected endogenous expression of *FOXC2-AS1* in blood cells of healthy individuals and LD patients. Furthermore, they found that, consistent with the results obtained in cancer cell lines, endogenous *FOXC2-AS1* positively regulates the expression of wild-type FOXC2 and its frameshift LD mutant in normal, non-cancer cells. So far, such regulation has only been demonstrated in peripheral blood cells; the next step would be to confirm the expression of *FOXC2-AS1* in LECs and study its regulatory role in lymphatic vascular development and homeostasis.

In summary, recent research has implicated lncRNAs in the regulation of several key molecular players and pathways involved in lymphangiogenesis (Table 1). This is a fast-moving area of research and some results still require validation in vivo before they can be considered fully reliable. Future studies in this emerging field should move beyond the predominantly in vitro work carried out so far and more thoroughly examine the functions of lymphatic-associated lncRNAs in animal models.

**Table 1:** Summary of the lymphatic-associated lncRNAs discussed in this review

Long noncoding RNA	Physiological or pathological role	Direct and downstream targets	Type of mechanism	Molecular function	Physiological or disease model	References
*AFAP1-AS1*	Oncogene	↑VEGF-C	Post-transcriptional	Acts as a sponge for miR-27b-3p	Cervical cancer	[116]
*ANRIL*	Promoter of diabetic wound healing	↑PROX1	Post-transcriptional	Acts as a sponge for miR‐181a	LEC-based in vitro model of diabetic wound healing	[167]
	Oncogene	↑VEGF-C, ↑VEGFR-3, ↑LYVE-1	Unknown	Unknown	Colorectal cancer	[165]
*ASLNC07322*	Oncogene	↑VEGF-C	Post-transcriptional	Acts as a sponge for miR-128-3p	Colon cancer	[114]
*BCYRN1*	Oncogene, activator of Wnt signaling	↑WNT5A ↑VEGF-C	Transcriptional/epigenetic	Recruits HNRNPA1 to the *WNT5A* promoter	Bladder cancer	[106]
	Oncogene, exosomal lncRNA	↑VEGFR-3	Post-transcriptional	Stabilizes *VEGFR-3* mRNA		
*BLACAT2*	Oncogene	↑VEGF-C	Transcriptional/epigenetic	Recruits WDR5 to the *VEGF-C* promoter	Bladder cancer	[102]
*circNFIB1*	Tumor suppressor, inhibitor of PI3K/AKT signaling	↑PIK3R1 ↓VEGF-C	Post-transcriptional	Acts as a sponge for miR-486-5p	Pancreatic ductal adenocarcinoma	[127]
*DANCR*	Oncogene	↑VEGF-C	Post-transcriptional	Acts as a sponge for miR-335	Bladder cancer	[120]
*ELNAT1*	Oncogene, exosomal lncRNA	↑SOX18, ↑PROX1	Transcriptional/epigenetic	Recruits HNRNPA1 to the *SOX18* promoter	Bladder cancer	[187]
*FOXC2-AS1*	Oncogene	↑FOXC2	Post-transcriptional	Stabilizes *FOXC2* mRNA	Osteosarcoma	[208]
					Colorectal cancer	[212]
					Gastric cancer	[213]
					Blood cells of healthy individuals and LD patients ^a^	[216]
*GAS5*	Promoter of diabetic wound healing	↑PROX1	Post-transcriptional	Acts as a sponge for miR-217	LEC-based in vitro model of diabetic wound healing	[172]
*HANR*	Oncogene	↑EAG1, ↑HIF-1α, ↑VEGF-A	Post-transcriptional	Acts as a sponge for exosomal miR‐296	Hepatocellular carcinoma	[142]
*HNF1A-AS1*	Oncogene, activator of PI3K/AKT signaling	↑PIK3CD, ↑VEGF-C	Post-transcriptional	Acts as a sponge for miR-30b-3p	Gastric cancer	[123]
*HUMT*	Oncogene	↑FOXK1, ↑HIF-1α , ↑VEGF-C	Transcriptional/epigenetic	Recruits YBX1 to the *FOXK1* promoter	Triple-negative breast cancer	[134]
*LncCCLM*	Tumor suppressor	↓IGF-1	Post-transcriptional	Promotes STAU1-mediated degradation of *IGF-1* mRNA	Cervical cancer	[149]
*LNMAT1*	Oncogene	↑CCL2, ↑VEGF-C	Transcriptional/epigenetic	Recruits HNRNPL to the *CCL2* promoter	Bladder cancer	[104]
*LNMAT2*	Oncogene, exosomal lncRNA	↑PROX1	Transcriptional/epigenetic	Recruits HNRNPA2B1 to the *PROX1* promoter	Bladder cancer	[180]
*LETR1*	Lymphatic lineage-specific lncRNA, gatekeeper of the LEC transcriptome	↓KLF4, ↑SEMA3C and others	Transcriptional/epigenetic	Recruits RBBP7 to target gene promoters	Cultured LECs vs BECs	[83]
*MAPK8IP1P2*	Tumor suppressor, activator of Hippo signaling	↑NF2, ↑RASSF1, ↑RASSF5	Post-transcriptional	Acts as a sponge for miR-146b-3p	Thyroid cancer	[193]
*MFSD4A-AS1*	Oncogene	↑VEGF-C	Post-transcriptional	Acts as a sponge for miR-30c-2-3p and miR-145-3p	Thyroid cancer	[121]
		↑VEGF-A		Acts as a sponge for miR-139-5p		
*MIAT*	Inducer of ADMSC differentiation into LECs	↑PROX1	Post-transcriptional	Acts as a sponge for miR-495	Cultured ADMSCs	[176]
*NEAT1*	Physiological activator of lymphangiogenic factors	↑FGF-1, ↑VEGF-A, ↑VEGF-C, ↑VEGF-D	Post-transcriptional (nuclear paraspeckle-associated) and translational	Promotes IRES-dependent mRNA translation	Normoxic and hypoxic mouse cardiomyocytes, mouse breast cancer cells	[146]
*VEGFC-LNC*	Endothelial-specific lncRNA	↑VEGF-C	Unknown	Unknown	Cultured HUVECs ^a^	[100]
*VESTAR*	Oncogene	↑VEGF-C	Post-transcriptional	Promotes HuR-mediated stabilization of *VEGF-C* mRNA	Esophageal squamous cell car-inoma	[110]

ADMSCs, adipose‑derived mesenchymal stem cells; AKT, protein kinase B; BECs, blood endothelial cells; CCL2, C-C motif chemokine ligand 2; FGF-1, fibroblast growth factor 1; FOXC2, forkhead box C2; FOXK1, forkhead box K1; HIF-1α, hypoxia-inducible factor 1 subunit alpha; HNRNPA1, heterogeneous nuclear ribonucleoprotein A1; HNRNPA2B1, heterogeneous nuclear ribonucleoprotein A2/B1; HNRNPL, heterogeneous nuclear ribonucleoprotein L; HUVECs, human umbilical vein endothelial cells; HuR, human antigen R; IGF-1, insulin-like growth factor 1; IRES, internal ribosome entry site; KLF4, Krüppel-like factor 4; LECs, lymphatic endothelial cells; PI3K, phosphoinositide 3-kinase; PIK3CD, phosphoinositide-3-kinase, catalytic subunit delta; PIK3R1, phosphoinositide-3-kinase, regulatory subunit 1; LYVE-1, lymphatic vessel endothelial hyaluronan receptor 1; NF2, neurofibromin 2; PROX1, Prospero homeobox 1; RASSF1, Ras association domain family member 1; RASSF5, Ras association domain family member 5; RBBP7, retinoblastoma binding protein 7; SEMA3C, semaphorin 3C; SOX18, SRY-box transcription factor 18; STAU1, Staufen double-stranded RNA binding protein 1; VEGF-A, vascular endothelial growth factor A; VEGF-C, vascular endothelial growth factor C; VEGF-D, vascular endothelial growth factor D; VEGFR-3, vascular endothelial growth factor receptor-3; WDR5, WD repeat-containing protein 5; WNT5A, Wnt family member 5A; YBX1, Y-box binding protein 1

^a^Physiological expression has so far been demonstrated only in non-lymphatic cell types

## LncRNAs as diagnostic tools and therapeutic targets in lymphatic vascular diseases and cancer

LncRNAs are emerging as promising targets for therapeutic intervention and as potential diagnostic biomarkers. The fact that some lncRNAs can be lineage-, tissue- or disease-specific [4] underscores their diagnostic potential. For example, the lncRNA prostate cancer antigen 3 (*PCA3*) is specifically overexpressed in prostate cancer compared to normal prostate tissue and has been approved as a diagnostic biomarker for the early detection of prostate cancer [217]. As the pioneering lncRNA biomarker, *PCA3* demonstrates a diagnostic accuracy superior to the most commonly used prostate-specific protein biomarker, prostate-specific antigen (PSA) [218]. In light of the above, the identification of *LETR1*, the first lymphatic lineage-specific lncRNA, paves the way for the development of novel diagnostic approaches to diseases involving the lymphatics such as lymphedema. Furthermore, lncRNAs associated with lymphatic pathologies, such as those promoting tumor lymphangiogenesis and lymphatic metastasis, may also have diagnostic and prognostic value.

LncRNAs represent promising therapeutic targets for the following reasons: (1) lncRNAs are generally expressed at lower levels than mRNAs [219] and hence require lower doses of potentially toxic drugs to achieve an equal therapeutic effect; (2) lncRNA expression can be lineage-, tissue-, disease-, or even cell population-specific [220], allowing for more selective therapy with fewer side-effects; (3) the fact that lncRNAs do not need to be translated into protein offers the advantage of greater drug design flexibility and faster response to therapy. As discussed above, the majority of lncRNAs involved in lymphatic-associated diseases constitute oncogenes promoting tumor lymphangiogenesis and lymphatic metastasis. These lncRNAs are therefore the most obvious targets for therapeutic intervention. Several therapeutic strategies to suppress oncogenic lncRNAs or alter their epigenetic effects are outlined in the paragraph below. Targeting lncRNAs could also be a promising therapeutic option for the treatment of other lymphatic vascular pathologies outside the cancer context. For example, downregulation of anti-lymphangiogenic lncRNAs or upregulation of pro-lymphangiogenic lncRNAs may improve delayed wound healing in diabetic patients and provide a therapeutic benefit in primary or secondary lymphedema, including postsurgical lymphedema after lymph node removal.

Since the advantages and limitations of various therapeutic strategies targeting lncRNAs have been extensively reviewed elsewhere [221–224], we will only briefly reintroduce them to the reader. These strategies include small-molecule inhibitors, RNA interference (RNAi), antisense oligonucleotides (ASOs), ribozymes, and genome editing tools. Small-molecule inhibitors bind to lncRNAs, changing their secondary structures or masking their protein-binding sequences. Alternatively, they may bind to RNA-binding proteins disrupting their interactions with lncRNAs. A variety of methods including small-molecule microarrays, label-based methods, mass spectrometry, dynamic combinatorial chemistry, NMR spectroscopy and virtual screening can be used to identify small molecule lncRNA binders [225]. At first glance, small-molecule lncRNA inhibitors appear to be a promising therapeutic option due to their high tissue penetration ability. However, if such molecules need to be delivered to the lymphatics, their small size becomes an issue because small molecules are readily reabsorbed into the bloodstream instead of accumulating in the lymphatic system [226]. A potential solution to this problem is the conjugation or physical encapsulation of lncRNA-targeting small molecules into various nanocarriers (see the section below on lymphatic delivery). The lncRNA-targeting strategy based on RNAi takes advantage of the natural process that largely occurs in the cytoplasm and employs small interfering RNAs (siRNAs) or short hairpin RNAs (shRNAs) as guides for sequence-specific gene silencing [227]. RNAi has been widely used to knock down predominantly cytoplasmic lncRNAs [228–231], making it a potential therapeutic approach. The main advantage of RNAi is its simplicity, as most cells already have functional RNAi machinery and do not require additional enzymatic components. On the other hand, dsRNA-induced immune responses, incomplete silencing, and widespread off-target effects limit the use of RNAi in the clinic [232–234]. A number of approaches are being explored to address these issues. For example, off-target effects have been minimized by increasing the length of siRNAs from the ‘conventional’ 21–23 nucleotides to 27 nucleotides and reducing siRNA concentrations to picomolar levels [235–237]. Furthermore, the ongoing development of novel algorithms and machine learning approaches for the analysis of siRNA-RNA interaction networks offers the potential to circumvent dsRNA-induced immune responses and select the most effective siRNAs for further clinical use [237–239]. ASOs are short, chemically synthesized, single-stranded antisense DNA oligonucleotides that bind to target lncRNA through base pairing rules, triggering RNase H-mediated lncRNA degradation [240]. Locked nucleic acid GapmeRs (LNA GapmeRs) are similar to native ASOs except that they are end-modified by LNA to increase nuclease resistance and binding affinity towards complementary lncRNA molecules [241]. Given their greater effectiveness in targeting nuclear transcripts compared to RNAi approaches [242], LNA GapmeRs have become the most widely used means of knocking down lncRNA in the nucleus. In addition, LNA GapmeRs show promise in relieving the repression of mRNAs by their natural antisense lncRNA transcripts (NATs). Commonly termed “antagoNATs”, these LNA GapmeRs inhibit the sense-antisense interaction between mRNAs and complementary NATs, triggering the NAT cleavage by RNase H and subsequent degradation by exonucleases [243]. Mixmers represent yet another type of chemically modified ASOs composed of alternating short stretches of LNA and DNA. In contrast to GapmeRs, mixmers do not trigger RNase H-dependent degradation of lncRNAs, acting instead as steric blockers of their targets [241]. ASOs have several advantages over siRNAs, including independence from the RNAi machinery, lower immunogenicity, and the ability to enter the nucleus more easily due to their small size [222]. Moreover, ASOs have greater specificity than siRNAs and cause fewer off-target effects [221]. Nonetheless, imperfect binding of ASOs to partially complementary regions in RNA, or to proteins, remains an issue, resulting in unintended off-target effects associated with hepato-, renal-, and neurotoxicity [244]. A possible solution to this problem is to train machine learning models to predict ASO toxicity using toxicity-associated sequence features as inputs [245]. Another lncRNA-targeting strategy involves ribozymes and deoxyribozymes, which are nucleic acid molecules with enzymatic activity that can be designed to target lncRNAs through base pairing, catalyzing their cleavage *in trans* [246, 247]. *Trans*-cleaving ribozymes, such as the hammerhead ribozyme (hhRz), have a significant advantage over exogenous ribonucleases for lncRNA cleavage because they are less likely to elicit a host immune response and are small enough to be easily incorporated into gene therapy vectors [248]. Furthermore, hhRzs have a low tolerance for even a single nucleotide mismatch with their targets [249], implying that they will have fewer off-target effects than siRNAs or ASOs. Indeed, when hhRz libraries were used for functional gene discovery, they caused less off-target effects than siRNA libraries [237]. The fact that hhRz has reached the stage of clinical trials for HIV-1 infection [250] demonstrates its potential for clinical applications, including lncRNA-targeted therapies. However, as with any technology, hhRzs have limitations. One of the concerns is that, despite their higher specificity, hhRzs have lower suppressive activity compared to siRNAs [237]. In the future, the discovery of more ribozyme motifs and a better understanding of the molecular and structural mechanisms underlying ribozyme action will likely lead to the engineering of new variants of *trans*-cleaving ribozymes with enhanced therapeutic properties [251]. Finally, genome-editing tools offer a range of opportunities not only for lncRNA inactivation but also for restoring the functions of pathologically downregulated or lost lncRNAs [222, 252]. These tools include, among others, zinc finger nucleases (ZFNs), transcription activator-like effector nucleases (TALENs), the clustered regularly interspaced short palindromic repeats (CRISPR/Cas9) system and its modifications such as base editing, prime editing, RNA editing with Cas7-11, CRISPR-based epigenetic editing and programmable addition via site-specific targeting elements (PASTE) [253–256]. The undisputed advantage of genome-editing tools for therapeutic targeting of lncRNAs is their high effectiveness. For example, a ZFN-based approach reduced the expression of the highly abundant lncRNA *MALAT1* in human lung cancer cells by more than 1000-fold, which is 50 times greater than the 20-fold reduction achieved with ASOs [257]. However, like any technology, genome-editing tools such as CRISPR/Cas9 come with their own caveats. Potential off-target effects limit their clinical application [258–260], albeit to a lesser extent than with traditional technologies like RNAi. The development of computational prediction algorithms and machine learning models [261, 262], as well as the engineering of nucleases with improved specificity [263], are among the approaches taken to address this issue. Furthermore, successful genome editing requires the delivery of a large cargo, such as long CRISPR/Cas9 sequences, into target tissues and cells, which remains a major therapeutic challenge [264]. The development of novel carrier systems for the delivery of large genome-editing agents remains an area of active research [265], and some of the proposed approaches are discussed in the next section. Overall, a number of promising strategies are currently being explored for therapeutic targeting of lncRNAs. Although the field is still developing and no effective therapy has yet been approved for clinical use, lncRNA-based therapeutics hold great potential for the treatment or even prevention of a variety of lymphatic-associated diseases, including metastatic cancer.

## Approaches to lymphatic delivery of lncRNA-based therapeutics

The choice of optimal delivery system is one of the most important and challenging problems in the development of therapies targeting lncRNAs in the lymphatic endothelium. Many current drugs that aim to reach the lymphatic system, such as anticancer chemotherapeutic agents, are administered intravenously. However, when administered in this way, these drugs not only show poor uptake into the lymphatics [266, 267] but also cause toxicity upon entering normal, unaffected organs and tissues. Moreover, they are quickly eliminated from the body via renal and hepatobiliary clearance pathways instead of accumulating in the lymphatic system [268]. The two primary reasons for this are the suboptimal route of administration and the small drug size of less than 5‒10 nm, which allows drug reabsorption back into the circulation through blood vessel walls [269, 270]. A more promising approach is to use the lymphatic vasculature as a delivery route and encapsulate drugs in nanocarriers with sizes ranging from 20 to 100 nm, which are thought to be optimal for lymphatic uptake and LN accumulation [268, 271]. Multiple strategies are currently being developed for the targeted delivery of therapeutic agents to the lymphatic vasculature. In these strategies, cargo molecules are packaged into a variety of liposomes, micelles, nanoparticles, and other nanocarrier systems [272] before being injected into subcutaneous or intradermal lymphatic vessels using conventional or microneedle techniques [226]. Though we discuss some nanocarrier delivery strategies in the following paragraphs, the literature on this subject is vast and rapidly growing, and we refer the reader to several comprehensive reviews for more information and references [226, 272–274]

As many times before in history, insights from nature may provide important clues for solving the problem of targeted lymphatic delivery. As discussed above, oncogenic lncRNAs can be loaded into lipid-based EVs (e.g., exosomes), secreted by cancer cells and then successfully internalized by other cells such as LECs (Fig. 3). This EV internalization mechanism can be modified to handle therapeutic molecules targeting lncRNAs. There are several reasons why endogenous or artificial EVs hold promise for the development of lncRNA-targeted therapies for various diseases involving the lymphatic vasculature. First, the payload in such vesicles is encapsulated in a lipid bilayer, reducing concerns over its stability. Second, lipid-based EVs are less immunogenic and more stable in vivo than the widely used viral vectors [223]. Third, artificial EVs coated with suitable hydrophilic polymers and/or assembled from several lipid components can achieve increased cellular uptake and circulatory half-life with reduced cytotoxicity [221]. Fourth, the low packaging capacity of endogenous exosomes can be markedly increased through integration with artificial liposomes or lipid nanoparticles. The resulting exosome-liposome hybrids can encapsulate molecules as large as CRISPR/Cas9 expression vectors [275]. However, most importantly, such exosome-liposome hybrids could retain specific ligands naturally targeting them to LECs. It is well known that LECs cannot be efficiently transfected with conventional cationic liposomes such as Lipofectamine 2000 or Oligofectamine, which lack the protein components of endogenous EVs. To overcome this problem, it will be necessary to identify the specific ligands on the surface of secreted EVs that are responsible for targeting them to LECs. One such ligand might be laminin γ2 because its knockdown in cancer cells results in the secretion of dysfunctional EVs which cannot be efficiently internalized by LECs and show a reduced ability to drain into LNs and promote lymphangiogenesis [276]. In addition, several surface determinants for endothelial targeting have been identified in the blood vascular endothelium [277]. It remains to be seen whether some of these determinants can be utilized for the delivery of therapeutic EVs into LECs. The successful identification of LEC-targeting ligands would allow engineering of artificial EVs that can deliver their therapeutic cargo via selective uptake by LECs. One way to achieve this is by expressing the targeting ligand in donor cells, which will then be passed through membrane pores or microfluidic devices [278] to form artificial exosomes with the targeting ligand on their surface. The desired payload, such as molecules selectively interfering with lncRNA expression, can then be loaded into these artificial exosomes via various physical or chemical methods [279]. Another avenue of research is genetic modification of cancer cells that naturally secrete EVs targeted to LECs. The payload of such EVs could be genetically modified or even replaced to serve a therapeutic purpose. The proof of concept for this approach was recently demonstrated with genetically engineered ovarian cancer cells stably overexpressing the tumor-suppressor miR-92b-3p. The engineered cells successfully packaged miR-92b-3p into exosomes and delivered the miRNA to human umbilical vein endothelial cells (HUVECs), exhibiting potent anti-angiogenic and anti-tumor activity in vitro and in vivo [280].

Lymphatics serve as a critical conduit for transporting dietary lipids from the gastrointestinal tract into systemic circulation. A typical route for intestinal fat absorption is the chylomicron pathway, which transports dietary lipids from enterocytes to lymphatics in the form of large triglyceride-rich lipoprotein particles known as chylomicrons [281, 282]. Another important route that allows particles such as pathogens to reach the intestinal lymphatics is the microfold cell (M cell) pathway. M cells are located within the intestinal epithelial layer in Peyer’s patches, groups of mucosa-associated lymphoid follicles mainly found in the ileum [283, 284]. Their primary function is to capture antigens from the intestinal lumen, actively transport them to the sub-mucosal lymphoid tissues, and present them to immune cells such as dendritic cells and lymphocytes [285, 286]. Therefore, M cells are increasingly viewed as a potential portal for oral drug delivery to the lymphatic system via the lymphatic capillaries surrounding Peyer’s patches [287–289]. These two pathways, especially the chylomicron pathway, could be utilized to deliver lipid nanoparticles containing therapeutic cargo to intestinal lymphatics and further to the lymph nodes. This strategy has potential to be developed into a method for the lymphatic delivery of therapeutic molecules targeting lncRNAs. For more details on the chylomicron-based, M cell-based and other lymphatic delivery strategies, we refer the reader to two recent comprehensive reviews [288, 290].

In addition to lipid-based EVs, several other types of delivery vectors have been proposed for lncRNA-targeting therapeutics [252]. They include various polymer-based nanoparticles and micelles, dendrimers, carbon nanotubes, and nanoparticles comprising metals or metalloids [221, 252]. Many of these nanoparticle formulations have already been successfully used for lymphatic delivery [290]. At first glance, these vectors might seem less promising because they lack LEC-specific targeting. However, this problem can be addressed by conjugating nanoparticles with LEC-specific homing molecules such as antibodies. For example, selective targeting to LECs has been achieved by conjugating polyethylene glycol-coated magnetic nanoparticles with a monoclonal antibody to lymphatic vessel endothelial hyaluronan receptor 1 (LYVE-1) [291], a selective marker of the lymphatic endothelium [292]. A similar strategy was recently employed for selective delivery of lipid nanoparticles containing small interfering RNA (siRNA) into LECs. The nanoparticles were conjugated with a monoclonal antibody to the LEC-specific marker podoplanin (PDPN) [293], thus providing an alternative to the above-discussed addition of targeting ligands to the particle surface. Furthermore, antibodies can be directly conjugated to therapeutic oligonucleotides such as siRNAs to facilitate targeted delivery to specific tissues and cell types without the use of a vector [294]. This approach to siRNA delivery into LECs, however, has a major limitation: the siRNA-antibody conjugates are often trapped in the endocytic compartment, thereby reducing their functional activity [295]. Selective targeting of therapeutic oligonucleotides or nanoparticles to LECs can be also achieved by conjugating them to aptamers directed against lymphatic markers such as LYVE-1, PDPN or VEGFR-3. Aptamers, also known as “chemical antibodies”, are single-stranded oligonucleotides selected for their affinity to a protein or other biomolecule of interest by a repetitive in vitro process called systematic evolution of ligands by exponential enrichment (SELEX) [296]. Since aptamers are 15 to 20 times smaller than antibodies, they offer superior normal tissue and tumor penetration [297] and therefore appear more promising than antibodies for the selective delivery of therapeutic molecules to specific sites in the body. Homing peptides represent yet another strategy for selectively targeting therapeutic molecules and nanocarriers to the lymphatics. They have, like aptamers, a much smaller molecular size than antibodies, which allows for better tissue penetration, faster clearance and lower immunogenicity [298]. The cyclic peptide Ly‐1P (CGNKRTRGC), first identified by Laakkonen et al. [299], represents a good example of such a homing peptide. Ly‐1P effectively targets tumor‐associated lymphatics, tumor cells and tumor-associated macrophages [299, 300], underscoring the promising clinical value of this lymphatic-homing peptide for imaging and therapy of tumors [301]. Furthermore, conjugation of Ly‐1P to nanoparticles [302], polymeric micelles [303] and liposomes [304] has been shown to facilitate the successful delivery of these nanocarriers to tumor lymphatics, resulting in the targeted release of anticancer drugs at metastatic tumor sites in vivo [303, 305]*.* Thus, the conjugation of lymphatic-homing peptides to nanocarriers may be a promising strategy for the lymphatic delivery of lncRNA-targeting therapeutics.

Despite the great potential of the above-discussed nanocarrier systems, viral vectors currently represent the most efficient means of delivering therapeutic nucleic acids into LECs. Several types of viral vectors such as genetically modified adenoviruses, adeno-associated viruses, retroviruses or lentiviruses are suitable for mediating RNA interference (RNAi) in multiple cell types including LECs. Each vector type has its advantages and disadvantages [306, 307] and should be selected in accordance with the therapeutic objective. The successful use of viral vectors to target lncRNAs has already been described in the literature—for example, knockdown of the lncRNA *HOTAIR* by lentiviral vector-mediated RNAi inhibited proliferation and invasion of endometrial carcinoma cells in vitro and in vivo [308]. Nevertheless, viral vectors are not without limitations. Innate and adaptive immune responses present obstacles to clinical application [309]; furthermore, viral vectors generally lack cell type specificity and may be difficult and costly to mass produce. In summary, a number of delivery systems based on lipid, polymeric or inorganic nanoparticles as well as viral vectors hold promise for the selective delivery of therapeutic molecules targeting lncRNAs to the lymphatic vasculature. Similar delivery systems could be used to target oncogenic lncRNAs in cancer cells, thereby inhibiting tumor lymphangiogenesis and lymphatic metastasis.

## Conclusion

To date, our understanding of the molecular mechanisms underlying lymphangiogenesis has been primarily obtained from analysis of various proteins and protein signaling cascades. Recent research has revealed that long non-coding RNAs, a heterogeneous and functionally diverse class of molecules, have a previously unsuspected regulatory role in this process. Two new subclasses of pro-lymphangiogenic and anti-lymphangiogenic lncRNAs are emerging as a novel avenue for manipulating the lymphatic vasculature. In the cancer setting, these two groups of lncRNAs act as oncogenes or tumor suppressors by promoting or inhibiting tumor lymphangiogenesis and lymphatic metastasis. Furthermore, there is now evidence for a third, lineage-specific group of lncRNAs that are specifically expressed in normal, non-cancerous lymphatic endothelium and are critically involved in the regulation of the lymphatic-specific molecular and cellular mechanisms. The discovery of the first lymphatic-specific lncRNA *LETR1* not only enhances our understanding of how lymphatic development and function are controlled, but also raises new questions. For example, the fact that *LETR1* acts as gatekeeper of the LEC transcriptome leaves us with the important question: how is *LETR1* itself regulated and is there a feedback loop between *LETR1* and its targets? Another interesting question is whether lymphatic-specific lncRNAs have a role in non-cancerous lymphatic pathologies such as lymphedema. While we have barely scratched the surface of the complex lncRNA-mediated mechanisms governing lymphangiogenesis, we are already beginning to understand the therapeutic and diagnostic potential of lncRNAs in a variety of lymphatic-associated diseases including metastatic cancer. At present, there is only limited experience of therapeutic targeting of lncRNAs and the underlying methodological concepts are still mainly at the laboratory stage. One problem will be to identify the optimal delivery system combining high efficacy with lymphatic selectivity. Yet, despite the many challenges, the next few decades are likely to see substantial advances in the treatment of lymphatic-associated diseases based on highly specific or even individualized lncRNA-targeted therapies.

## Data Availability

Not applicable.
